# A Comparison of Blood Pathogen Detection Among Droplet Digital PCR, Metagenomic Next-Generation Sequencing, and Blood Culture in Critically Ill Patients With Suspected Bloodstream Infections

**DOI:** 10.3389/fmicb.2021.641202

**Published:** 2021-05-17

**Authors:** Bangchuan Hu, Yue Tao, Ziqiang Shao, Yang Zheng, Run Zhang, Xuejing Yang, Jingquan Liu, Xi Li, Renhua Sun

**Affiliations:** ^1^Intensive Care Unit, Zhejiang Provincial People’s Hospital, People’s Hospital of Hangzhou Medical College, Hangzhou, China; ^2^The Laboratory of Pediatric Infectious Diseases, Pediatric Translational Medicine Institute, Shanghai Children’s Medical Center, Shanghai JiaoTong University School of Medicine, Shanghai, China; ^3^Centre of Laboratory Medicine, Zhejiang Provincial People’s Hospital, People’s Hospital of Hangzhou Medical College, Hangzhou, China

**Keywords:** droplet digital PCR, bloodstream infection, blood culture, pathogen, metagenomic next-generation sequencing

## Abstract

Metagenomic next-generation sequencing (mNGS) and droplet digital PCR (ddPCR) have recently demonstrated a great potential for pathogen detection. However, few studies have been undertaken to compare these two nucleic acid detection methods for identifying pathogens in patients with bloodstream infections (BSIs). This prospective study was thus conducted to compare these two methods for diagnostic applications in a clinical setting for critically ill patients with suspected BSIs. Upon suspicion of BSIs, whole blood samples were simultaneously drawn for ddPCR covering 20 common isolated pathogens and four antimicrobial resistance (AMR) genes, mNGS, and blood culture. Then, a head-to-head comparison was performed between ddPCR and mNGS. A total of 60 episodes of suspected BSIs were investigated in 45 critically ill patients, and ddPCR was positive in 50 (83.3%), mNGS in 41 (68.3%, not including viruses), and blood culture in 10 (16.7%) episodes. Of the 10 positive blood cultures, nine were concordantly identified by both mNGS and ddPCR methods. The head-to-head comparison showed that ddPCR was more rapid (~4 h vs. ~2 days) and sensitive (88 vs. 53 detectable pathogens) than mNGS within the detection range of ddPCR, while mNGS detected a broader range of pathogens (126 vs. 88 detectable pathogens, including viruses) than ddPCR. In addition, a total of 17 AMR genes, including 14 *bla_KPC_* and 3 *mecA* genes, were exclusively identified by ddPCR. Based on their respective limitations and strengths, the ddPCR method is more useful for rapid detection of common isolated pathogens as well as AMR genes in critically ill patients with suspected BSI, whereas mNGS testing is more appropriate for the diagnosis of BSI where classic microbiological or molecular diagnostic approaches fail to identify causative pathogens.

## Introduction

Bloodstream infection (BSI) is a frequent and life-threatening complication in critically ill patients, often leading to septic shock and death (Prowle et al., 2011; Adrie et al., 2017). These unwanted clinical outcomes are commonly caused by the inability to rapidly and accurately diagnose causative pathogens at the onset of illness when antibiotic treatment is most effective (Kumar et al., 2006). As reported in septic patients within the first 6 h of documented hypotension, every 1-h delay in the use of effective antibiotics leads to an average decrease in the survival rate by 7.6%, and the survival rate of severe sepsis drops from 80 to 10% if appropriate therapy is not given within 24 h (Kumar et al., 2006; Ferrer et al., 2014). For optimal treatment efficacy, the current guidelines recommend the initiation of broad-spectrum antimicrobial therapy as early as possible and preferably within 1 h of sepsis/septic shock recognition (Rhodes et al., 2017). However, in actual clinical practice, approximately 46% of empirical antibiotic treatments were shown to be inappropriate, leading to an increase in the mortality rate of 35%, and approximately 50% were either unnecessary or broad-spectrum antibiotics, increasing the risk of antibiotic resistance and toxicity (Paul et al., 2010; Campion and Scully, 2018). Therefore, rapid and accurate detection of pathogens for BSI is exceedingly valuable in directing early antimicrobial therapy, enabling better antibiotic stewardship, and further improving clinical outcomes.

Currently, blood culture coupled with antimicrobial susceptibility testing (AST) remains the gold standard for BSI diagnosis, as it is easy to perform and displays a good analytical performance. However, blood culture is limited by a long turnaround time, relatively low sensitivity (because of a low microbial inoculum or growth inhibition by prior use of antibiotics; Lamy et al., 2016; Pilecky et al., 2019), and low specificity (Liesenfeld et al., 2014; Opota et al., 2015). Although real-time quantitative (qPCR), microarray technology, nanoparticle-based assays, and sequencing can shorten the turnaround time to hours (Walker et al., 2016; van den Brand et al., 2018; Zhang et al., 2018; Dubourg et al., 2019), they are often not sensitive enough to detect bacteria at low concentrations. Recently, metagenomic next-generation sequencing (mNGS) and droplet digital PCR (ddPCR) have shown great potential in pathogen detection for patients with suspected BSIs. mNGS is culture-free and can analyze the entire microbial community in a clinical sample (Miao et al., 2018; Blauwkamp et al., 2019). Earlier studies have shown that mNGS can reliably detect pathogens from patients with febrile illness (Wylie et al., 2012), respiratory and gastric/digestive infections (Joensen et al., 2017; Mizrahi et al., 2017), and acute encephalitis/encephalopathy (Kawada et al., 2016). In addition, mNGS can help identify causative microorganisms for patients with suspected BSIs with high sensitivity and specificity (Gyarmati et al., 2016; Brenner et al., 2018; Goggin et al., 2019). ddPCR, as an emerging versatile tool with high sensitivity and excellent accuracy and precision, has been increasingly used in multiple clinical scenarios including BSIs (Li et al., 2018; Abram et al., 2020; Wouters et al., 2020). For example, a one-step, high-throughput ddPCR has been reported with high sensitivity (10 CFU per ml) and short assay time (less than 1 h) for rapid detection of low-abundance bacteria in patients with BSIs (Abram et al., 2020). However, the utility of mNGS and ddPCR for suspected BSIs in critically ill patients at ICUs has not been systematically evaluated.

In the present study, with a cohort of septic patients with suspected BSIs, we therefore comprehensively evaluated the clinical application of mNGS-based and ddPCR-based methods for rapid and accurate detection of pathogens, as well as antimicrobial resistance genes, and further performed a head-to-head comparison between mNGS-based and ddPCR-based methods against traditional blood culture as the gold standard.

## Materials and Methods

### Patients

This study was conducted in the general intensive care unit of Zhejiang Provincial People’s Hospital from April 1, 2019 to March 30, 2020. The patients suspected of having BSIs, aged greater than 18 years, were consecutively recruited from April 1 to December 21, 2019 in this study, and the remaining 3 months of the study were dedicated to molecular detection and data analysis. Clinical suspicion of BSI was determined by the development of organ dysfunction due to a dysregulated host response to infection, clinical signs, and symptoms. Suspected BSI was defined if the patient had a sudden high fever (*T* ≥ 38.5°C) accompanied by hemodynamic instability that could not be explained by a site-specific infection at another body site. In addition, the recruited patient also presented life-threatening organ dysfunction with an increase of two points or more in the sepsis-related organ failure assessment score. Organ dysfunction and the severity of illness were daily assessed by the sequential organ failure assessment (SOFA) and acute physiology and chronic health evaluation II (APACHE II) scoring systems, respectively. The exclusion criteria were (1) pre-existing BSI during hospitalization, (2) advanced cancer, or (3) any terminal-stage disease. The study protocol was approved by the Institutional Review Board and Ethics Committee (No. 2019KY002) of Zhejiang Provincial People’s Hospital, and all patients or their legal representatives gave informed written consent according to the ethics rules.

### Blood Culture and Pathogen Identification

Upon clinical suspicion of BSI, whole blood samples were simultaneously obtained for blood culture and molecular diagnosis. Two sets of blood cultures were collected for each patient according to routine clinical practice (Miller et al., 2018), and each set consisted of an aerobic bottle and an anaerobic bottle. The blood cultures were incubated at 37°C in a BacT/ALERT® 3D System (bioMérieux, France). When the system showed a positive signal, Gram staining was performed, followed by subculture on a Columbia blood agar plate at 37°C with 5% CO_2_. Following overnight incubation, the pathogens were further identified by matrix-assisted laser desorption-ionization time-of-flight mass spectrometry (MALDI-TOF MS; VITEK® MS system, bioMérieux, France).

### Antibiotic Susceptibility Test and Resistance Gene Detection

AST was performed using the commercial automated VITEK2 COMPACT system (bioMérieux, France) following the manufacturer’s protocols. The results for AST were interpreted according to the Clinical and Laboratory Standards Institute guidelines (CLSI, 2018). All of the fungi were also tested for *in vitro* susceptibilities to six antifungal agents, namely, caspofungin, fluconazole, itraconazole, voriconazole, 5-flucytosine, and amphotericin B, using broth microdilution methods, and the results were interpreted according to CLSI standards (CLSI, 2012). *Staphylococcus aureus* ATCC 25923, *Enterococcus faecalis* ATCC 29212, *Escherichia coli* ATCC 25922, and *Candida krusei* ATCC 6258 were used as reference strains to ensure reproducibility of the AST procedure.

In addition, carbapenemase-encoding resistance genes (*bla_KPC_*, *bla_IMP_*, *bla_NDM_*, *bla_VIM_*, and *bla_OXA_*) were also further screened by PCR (Jin et al., 2018).

### Plasma DNA Extraction and Droplet Digital PCR

Peripheral venous blood (5 ml) was drawn from each patient in an ethylenediaminetetraacetate (EDTA)-containing tube. Plasma was immediately isolated after centrifugation at 1,600×*g*, and 22°C for 20 min. Plasma DNA extraction was completed within 1 h from 2 ml of plasma using a Magnetic Serum/Plasma DNA Kit (TIANGEN Biotech, Beijing, China) and the Auto-Pure20B Nucleic Acid Purification System (Hangzhou Allsheng Instruments Company, Hangzhou, China) following the manufacturer’s protocol (Nie et al., 2019). DNA was eluted in 50 μl of elution buffer and used for ddPCR assay in a timely manner on the same day. The remaining DNA was stored at −80°C for further mNGS analysis.

Pathogens and AMR genes were detected using six assay panels (four for bacteria, one for fungi, and one for AMR genes) with a five-channel fluorescence ddPCR system (Pilot Gene Technology Company, Hangzhou, China). The target pathogens or AMR genes detected in this study are shown in Supplementary Table 1. ddPCR analysis was performed using a Pilot Gene Droplet Digital PCR System following the manufacturer’s protocol. Briefly, the ddPCR master mix for each testing panel had a final volume of 15 μl and contained 1x ddPCR premix, 1 μM forward and reverse primers, 300 nM each probe, 5 μl of isolated plasma DNA, and DNase-free water. After PCR amplification, droplets were analyzed for 35 min using an iScanner 5 chip scanner. Data analysis for droplet counts and amplitudes was performed with 30 min of hands-on time using GenePMS software version v2.0.01.20011. The synthesized DNA fragment was used as an internal control, and DNase-free water or blood samples from three healthy subjects were spiked with the internal control to monitor for external or reagent microbial contamination and cross-sample contamination.

### Metagenomic Next-Generation Sequencing and Analysis

Briefly, the same plasma DNA was quantified using a Qubit dsDNA HS Assay Kit (Life Technologies, CA, United States). DNA libraries were constructed by a tagmentation method in which fragmented DNA and partial adapters were added in 5 min and prepared from 2 ng of plasma DNA using a Nextera XT Library Preparation Kit (Illumina, CA, United States) following the manufacturer’s protocol. The quality of the libraries was assessed by a High-Sensitivity DNA Kit using an Agilent 2,100 Bioanalyzer (Agilent Technologies, Santa Clara, CA, United States), and quantitative PCR was used to quantify the DNA library before sequencing. Sequencing was performed on a NextSeq sequencer (Illumina, CA, United States), resulting in 15–20 million 75-bp single-end reads, on average, for each sample.

NGS data analysis was performed by using IDseqTM commercial bioinformatic pipeline (Vision Medicals, Shanghai, China). Raw data were preprocessed by the removal of low-quality reads and short reads (length <35 bp), low-complexity reads, as well as adapter trimming. Then, the reads aligned to the human reference genome (GRCh38) using Burrows-Wheeler Alignment (BWA) or derived from plasmids were also excluded from further analysis. Subsequently, the remaining reads for taxonomic assignment were performed by aligning against the curated microbial databases consisting of bacteria, viruses, and fungi, and the classification reference databases for microbial genome sequence were downloaded from the National Center for Biotechnology Information Reference Sequence (RefSeq) database (release version 68)^1^ comprising 35,749 bacterial and 4,340 viral genomes complemented by 16 selected fungal genomes.

Ten plasma samples from healthy subjects, who had no clinical manifestations of infection and were not receiving any antimicrobial medication at the time of blood collection, were sequenced to identify the reference ranges for pathogenic microorganisms including the ranges of mapped reads, coverage rates, and unique mapped reads. The presence of the pathogenic microorganisms was determined mainly based on the reference range of coverage rate and the number of unique mapped reads. The criteria for positive mNGS results of bacteria and virus are as follows: mNGS identified a microbe (species level) whose coverage rate scored 10-fold greater than that of any other microbes; fungi: mNGS identified a microbe (species level) whose coverage rate scored 5-fold higher than that of any other fungus because of its low biomass in DNA extraction as previously described (Miao et al., 2018). The microorganisms sorted into the top 10 list by the coverage rate were obtained for further analysis. Among the top 10 microorganisms, pathogenic microorganisms should be considered positive if the number of unique mapped reads was >3 and exceeded the upper limit of the reference range, and the coverage rate also exceeded the upper limit of the reference range. DNase-free water as a negative control was processed and sequenced in parallel for each sequencing run for contamination control and background control.

### Statistical Analysis

SAS 9.13 (SAS Institute, NC, United States) was used for database management and statistical analyses. Continuous variables were expressed as the mean and SD or median and interquartile range (IQR) where appropriate. The *t* test was used to analyze normally distributed continuous variables, whereas the Mann-Whitney *U* test was used to analyze nonnormally distributed continuous variables. Categorical variables were reported as frequencies and percentages and analyzed using the chi-square test. Value of *p* less than 0.05 were considered statistically significant.

## Results

### Characteristics of the Patients

A total of 45 patients suspected of having BSIs, including 35 men (77.8%), were consecutively recruited in the present study, of whom 13 had repeated blood collection for blood culture and molecular detection. The median age of the patients was 69 years (IQR, 55–76 years). For inflammatory markers, the mean plasma levels of C-reactive protein and procalcitonin were 148.1 mg/L (IQR, 84.1–233.8 mg/L) and 3.30 μg/L (IQR, 1.29–15.1 μg/L), respectively. The mean values (±SDs) for the SOFA and APACHE II scores were 10.8 ± 5.09 and 24.0 ± 8.95, respectively. Among these 45 patients with a 28-day mortality rate of 60.0%, 19 (42.2%) had acute kidney injury, 13 (28.9%) received renal replacement therapy, and 40 (88.9%) received mechanical ventilation. In addition, 37 patients (82.2%) were treated with vasopressors, and 41 (91.1%) received antibiotic therapy prior to blood culture. The overall average time for the ddPCR assay was 4.2 ± 0.51 h, which was significantly shorter than that for mNGS testing (49.3 ± 6.8 h, *p* < 0.01) and traditional blood culture (90.6 ± 10.9 h, *p* < 0.01; Table 1). Compared to those with negative detection, the patients with positive pathogen detection by blood culture, ddPCR, or mNGS assay had higher levels of plasma C-reactive protein (*p* = 0.014) and procalcitonin (*p* = 0.023) and lower systolic blood pressure (*p* = 0.05; Supplementary Table 2).

**Table 1:** Characteristics of the patients.

Clinical characteristics	*n* = 45
Age (years)	65.7 ± 13.8
Male, *n* (%)	37 (82.2)
Use of vasoactive drugs, *n* (%)	32 (71.1)
Norepinephrine, *n* (%)	31 (68.8)
Epinephrine, *n* (%)	4 (8.89)
Vasopressin, *n* (%)	12 (26.7)
Mechanical ventilation, *n* (%)	41 (91.1)
Acute kidney injury, *n* (%)	19 (42.2)
Renal replacement therapy, *n* (%)	13 (28.9)
Received antibiotic therapy prior to blood culture, *n* (%)	41 (91.1)
Overall average time for ddPCR detection (hours)	4.2 ± 0.51
Overall average time for mNGS detection (hours)	49.3 ± 6.8
Overall average time for blood culture coupled with antibiotic susceptibility testing (hours)	90.6 ± 10.9
**Physical examination findings**	
Temperature (°C)	38.2 ± 0.93
Systolic blood pressure (mmHg)	91.5 ± 16.7
Diastolic blood pressure (mmHg)	48.7 ± 11.3
**Complete blood counts and blood biochemistry**	
Platelet count, median (IQR) × 10^3^/μl	79.5 (37.0–184.5)
White blood cell count, median (IQR) × 10^3^/μl	10.8 (7.4–16.6)
C reactive protein (mg/L), median (IQR)	148.1 (84.1–233.8)
Procalcitonin (μg/L), median (IQR)	3.30 (1.29–15.1)
Serum creatinine (μmol/L), median (IQR)	121.0 (80.1–178.5)
Serum lactate (mmol/L), median (IQR)	2.15 (1.60–5.25)
SOFA score	10.8 ± 5.09
APACHE II score	24.0 ± 8.95
28-day mortality, *n* (%)	27 (60.0)

IQR, interquartile range; SOFA, sequential organ failure assessment; APACHE II, acute physiology and chronic health evaluation II; ddPCR, droplet digital PCR; mNGS, metagenomic next-generation sequencing. Values are presented as the mean ± SD, median (IQR), or number of subjects (percentage of the column total).

### Pathogens Detected by Blood Culture

In a total of 60 blood samples from 45 patients, blood culture detected 10 positives (16.7%), with seven positives for Gram-negative bacteria, one for Gram-positive bacteria, and two for fungi. Among the seven strains of Gram-negative bacteria, five pathogens were identified as *Klebsiella pneumoniae*, one as *Acinetobacter baumannii*, and one as *Bacteroides thetaiotaomicron*. Of these five strains of *K. pneumoniae*, two were carbapenem-resistant. In addition, the Gram-positive bacteria strain was *a* methicillin-sensitive *S. aureus*, and two strains of fungi were *Candida parapsilosis* and *Candida tropicalis*, which were both sensitive to triazoles and caspofungin (Table 2).

**Table 2:** Comparison of pathogen detection among the ddPCR, mNGS, and blood culture methods in patients with positive blood culture.

Sample	Blood culture	ddPCR	mNGS
5	*S. aureus*	*S. aureus*	*S. aureus*
		*S. epidermidis*	
		*E. faecium*	
			*Human gammaherpesvirus 4*
			*Human betaherpesvirus 6B*
9	*K. pneumoniae*	*K. pneumoniae*	
			*Human betaherpesvirus 5*
16	B. thetaiotaomicron^*^		*B. thetaiotaomicron*
		*A. baumannii*	*A. baumannii*
			*Human gammaherpesvirus 4*
22	*K. pneumoniae*	*K. pneumoniae*	*K. pneumoniae*
		*A. baumannii*	*A. baumannii*
			*S. maltophilia*
			*Human betaherpesvirus 5*
			*Human gammaherpesvirus 4*
28	*A. baumannii*	*A. baumannii*	*A. baumannii*
		*E. cloacae*	*E. cloacae*
		*C. albicans*	*C. albicans*
36	*C. parapsilosis*	*C. parapsilosis*	*C. parapsilosis*
			*Torque teno virus*
41	*C. tropicalis*	*C. tropicalis*	*C. tropicalis*
		*A. baumannii*	*A. baumannii*
		*K. pneumoniae*	
			*Human betaherpesvirus 5*
			*Human alphaherpesvirus 1*
49	*K. pneumoniae*	*K. pneumoniae*	*K. pneumoniae*
		*C. parapsilosis*	*C. parapsilosis*
		*P. aeruginosa*	
	*Carbapenemase-resistant*	*bla_KPC_* gene	
			*Human parvovirus B19*
			*Human betaherpesvirus 5*
54	*K. pneumoniae*	*K. pneumoniae*	*K. pneumoniae*
		*P. aeruginosa*	
		*S. hominis*	
	*Carbapenemase resistant*	*bla_KPC_* gene	
			*Human parvovirus B19*
			*Human betaherpesvirus 5*
			*Human betaherpesvirus 6B*
61	*K. pneumoniae*	*K. pneumoniae*	*K. pneumoniae*
		*E. faecium*	*E. faecium*
			E. avium^*^

ddPCR, droplet digital PCR; mNGS, metagenomic next-generation sequencing.

* *B. thetaiotaomicron*, *Enterococcus avium*, and viruses were out of the ddPCR detection range.

### Pathogens and AMR Genes Detected by ddPCR

**Figure 1:**
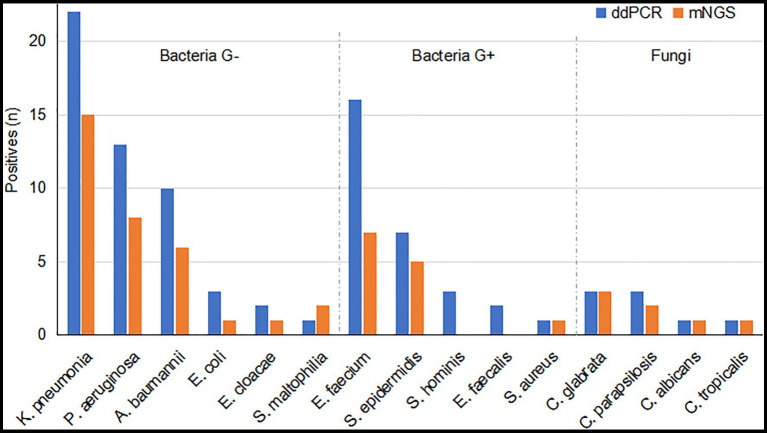
Pathogens detected by droplet digital PCR (ddPCR) and metagenomic next-generation sequencing (mNGS) within the detection range of ddPCR. Blue bars, pathogens detected by the ddPCR method; orange bars, pathogens detected by the mNGS method. ddPCR, droplet digital PCR; mNGS, metagenomic next-generation sequencing; G+ bacteria, Gram-positive bacteria; G− bacteria, Gram-negative bacteria.

Within the detection range of ddPCR, a total of 88 pathogens in 50 blood samples (83.3%) were detected by ddPCR. Among the 55 strains of Gram-negative bacteria detected, the top three bacteria were *K. pneumoniae* (*n* = 22), *Pseudomonas aeruginosa* (*n* = 13), and *A. baumannii* (*n* = 10), while the most detected bacteria in 29 strains of Gram-positive bacteria were *Enterococcus faecium* (*n* = 16) and *Staphylococcus epidermidis* (*n* = 7). In addition, the four fungal pathogens detected by ddPCR were *Candida albicans* (*n* = 1), *Candida glabrata* (*n* = 2), and *C. parapsilosis* (*n* = 1; Figure 1).

In addition to pathogen identification, four AMR genes were detected using the PilotAMR-1 panel, which targeted *bla_KPC_*, *mecA*, *vanA*, and *vanB*. Only the *bla_KPC_* and *mecA* genes were identified as positive by ddPCR in the present study (Table 3). The *bla_KPC_* gene was detected in 14 blood samples positive for *K. pneumoniae* (*n* = 7), *P. aeruginosa* (*n* = 2), *A. baumannii* (*n* = 1), and *K. pneumoniae* and *P. aeruginosa* (*n* = 4). The *mecA* gene together with *S. epidermidis* was identified in three blood samples. For 17 samples positive for AMR genes, the ddPCR assay and blood culture were concordantly positive in two samples, which were confirmed to carry *bla*_KPC-2_ gene by PCR method. However, none of the targeted pathogens were detected positive in DNase-free water and three blood samples from healthy subjects.

**Table 3:** AMR genes detected by ddPCR and the related pathogens.

AMR gene	Pathogens	Counts
*bla_KPC_*	*A. baumannii*	1
	*K. pneumoniae*	7
	*P. aeruginosa*	2
	*K. pneumoniae and P. aeruginosa*	4
*mecA*	*S. epidermidis*	3

AMR genes, antimicrobial resistance genes.

### Pathogens Detected by mNGS

A total of 126 pathogens were detected by mNGS, of which 53 were within the target pathogen range of the ddPCR assay. Of these 53 positives, the most frequently detected Gram-negative bacteria were *K. pneumoniae* (*n* = 17), *A. baumannii* (*n* = 9), and *P. aeruginosa* (*n* = 8), and the most common Gram-positive bacteria were *E. faecium* (*n* = 7) and *S. epidermidis* (*n* = 6). In addition, four fungi detected by ddPCR were also identified by mNGS (Figure 1).

Of the 73 pathogens found to be out of the detection range by the ddPCR assay, 65 positives were 11 species of viruses, five Gram-negative bacteria, two Gram-positive bacteria, and one fungus (Supplementary Figure 1). *Cytomegalovirus* (CMV; *n* = 23), *Torque teno virus* (*n* = 13), and *Epstein-Barr virus* (EBV; *n* = 11) were the most commonly detected viruses. In addition, *Pneumocystis jirovecii* was detected by mNGS testing in a sample. However, no pathogens were detected in DNase-free water as a negative control by mNGS. Of 10 blood samples from healthy subjects, EBV was detected in a sample by mNGS testing, but this case had no clinical symptoms and signs of BSI.

### Comparison Between ddPCR and mNGS

Within the target pathogen range of ddPCR, ddPCR detected 88 pathogens whereas mNGS detected 53 pathogens. A total of 51 positives were concordantly detected by ddPCR and mNGS. The 37 discordant positives detected only by the ddPCR assay were *E. faecium* (*n* = 10), *K. pneumoniae* (*n* = 8), *P. aeruginosa* (*n* = 5), *A. baumannii* (*n* = 4), *Staphylococcus hominis* (*n* = 3), *S. epidermidis* (*n* = 2), *E. coli* (*n* = 2), *E. faecalis* (*n* = 2), and *Enterobacter cloacae* (*n* = 1), whereas the two discordant positives detected only by mNGS were *Stenotrophomonas maltophilia* and *E. faecium* (Figure 1).

### Comparison Between ddPCR and mNGS Among Positive Blood Cultures

Among 10 positive blood cultures, nine samples were concordantly positive by blood culture and ddPCR, and one strain of *B. thetaiotaomicron* isolated only by blood culture was out of the ddPCR detection range. Of these nine positives, seven samples were identified as polymicrobial infections by the ddPCR assay, and overall, ddPCR detected 13 more pathogens compared with blood culture. Similarly, mNGS and blood culture were concordantly positive in nine samples. One discordant sample (Table 3, sample no. 9) was identified as *K. pneumoniae* by both blood culture and ddPCR, but detected negatively by mNGS. Conversely, among these nine positive samples, 23 additional pathogens were identified only by mNGS and not by blood culture, including 14 viruses, five Gram-negative bacteria, two Gram-positive bacteria, and two fungi.

Likewise, in the head-head comparison of ddPCR and mNGS, 15 positives were concordantly detected based only on the coverage of bacteria and fungi. The seven positives detected only by ddPCR were two *K. pneumoniae*, two *P. aeruginosa*, one *E. faecium*, one *S. hominis*, and one *S. epidermidis*, while the three positives detected only by mNGS were *S. maltophilia*, *B. thetaiotaomicron*, and *Enterococcus avium*. Notably, the latter two pathogens were not included by the ddPCR assay panels.

## Discussion

The present study compared the methods dedicated to early BSI detection, including ddPCR, mNGS, and blood culture, in critically ill patients with suspected BSI. The positivity rates were 68.3% (not including viruses) for mNGS and 83.3% for ddPCR, which were significantly higher than the 16.7% for blood culture. Of the10 positive blood cultures, the concordance between ddPCR and blood culture was 90%, equivalent to that between mNGS and blood culture.

Our findings were in line with the results of several previous studies that investigated the difference in the ability to detect pathogens from whole blood using nucleic acid-based and culture-based methods (Long et al., 2016; Korber et al., 2017; Grumaz et al., 2019). In a prospective, observational, single-center study including 256 plasma samples of 48 septic patients, Grumaz et al. (2019) reported that blood culture positivity was 11% and next-generation sequencing positivity was 71% over the whole study period. In a cohort of 398 critically ill patients with 470 episodes, Korber et al. (2017) found that the concordance between multiple real-time PCR (SeptiFast) and blood culture was 85.5%. Among a total of 120 relevant microorganisms identified, the positivity rate was 81.7% for SeptiFast and merely 52.5% for blood culture (Korber et al., 2017). In addition, in 78 plasma samples from ICU patients, the overall diagnostic sensitivity significantly increased from 12.8% (10/78) by blood culture alone to 30.8% (24/78) by NGS for ICU patients (Long et al., 2016). Several reasons could, to a certain extent, account for the lower positivity rate of blood culture than of ddPCR or mNGS methods. The false-negative blood culture might be attributable, at least in part, to the extremely low level of pathogens in the blood or prior antimicrobial therapy in a large majority of patients. Indeed, in the present study, 91.1% of the patients received antimicrobial therapy prior to blood culture. In addition, blood cultures lack sensitivity, especially for slowly growing, fastidious, or uncultivable microorganisms and fungi. In contrast, the molecular-based methods target pathogen nucleic acids, which are presumed to be present in the bloodstream (Lu et al., 2018; Papadopoulos, 2020). Therefore, these methods would be less affected by the survival status of pathogens and could exhibit much higher positive rates than culture-based methods.

This study was novel in investigating a head-to-head comparison of ddPCR and mNGS for detecting blood-borne pathogens in patients with suspected BSIs. In the present study, overall, the total number of pathogens detected by plasma DNA mNGS (*n* = 126) was significantly greater than that detected by ddPCR (*n* = 88). However, within the target pathogen range based on the ddPCR assay, the ddPCR method showed a higher detection rate of blood pathogens than the mNGS assay (88 positives in ddPCR vs. 53 positives in mNGS). Similar to our results, other studies have also reported that some common pathogenic bacteria detected by blood culture were occasionally missed by mNGS (Rossoff et al., 2019; Hogan et al., 2020), suggesting that plasma DNA mNGS testing still needs to be optimized to improve its sensitivity. Since the same plasma DNA samples were used for these two methods, in addition to the difference in the amount of template DNA used, the higher detection rate of ddPCR assay could also, in part, be explained by the low detection limit of ddPCR.

In the present study, a number of pathogenic microorganisms, including CMV (*n* = 23), Torque teno virus (*n* = 13), and EBV (*n* = 11), were missed by ddPCR but identified by plasma DNA mNGS. Therefore, these positive results for nucleic acids of viruses or rare pathogens posed interpretational challenges, and the causative pathogens should be cautiously differentiated based on mNGS testing in conjunction with clinical evaluation for guiding antimicrobial therapy for BSIs. Additionally, the real-world clinical impact of mNGS remains controversial. A single-center study including 79 patients from the Children’s Hospital of Chicago showed that mNGS testing was deemed to provide clinically relevant information in 80% of the positive tests (Rossoff et al., 2019). Nevertheless, two recent studies found that mNGS testing had limited value for patient care. Among a total of 82 plasma mNGS tests from pediatric and immunocompromised patients, Hogan et al. (2020) reported a positivity rate of 61%, but a positive clinical impact was identified in only 7.3% of cases. The low clinical impact proportion in the aforementioned study was close to that reported for mNGS on cerebrospinal fluid in a previous study where 8/204 (3.9%) tests led to a positive clinical impact (Wilson et al., 2019). Therefore, the application of mNGS in routine clinical practice requires more sophisticated interpretation of the results because the detection results may potentially complicate clinical decision-making.

Accurate identification of AMR in bacteria is essential for the proper administration of appropriate antibacterial agents. However, the total turnaround time of the current antibiotic resistance detection process for blood samples from patients with BSI requires 2 or 3 days, thus driving the need for advanced methods to rapidly identify AMR genes in bacterial pathogens. The present study demonstrated that four common target AMR genes, *bla_KPC_*, *mecA*, *vanA*, and *vanB* in one assay panel could also be detected directly from whole blood by ddPCR, and the detection results were reported in approximately 4 h. Notably, the timely and simultaneous identification of AMR genes and microbial/polymicrobial sources of infection can improve patient outcomes and allow enhanced monitoring of resistance mutations. To this end, Abram et al. (2020) recently reported a rapid diagnostic platform that integrates a novel one-step blood ddPCR assay and a high-throughput 3D particle counter system that could be simultaneously applicable for direct detection of a wide range of causative pathogens and AMR genes from whole blood specimens. Overall, the evidence presented in our study and the supporting literature suggests that the ddPCR assay alone or combined with other methods can provide a powerful platform for rapid diagnosis of BSIs and early initiation of appropriate antimicrobial therapy. However, to avoid false-negative results caused by the relatively narrow detection range of ddPCR, additional detection panels are needed. In contrast, the AMR detection for BSIs is not easily addressed by mNGS alone mainly due to low target abundance in blood samples and high background derived from the host.

Additionally, it is worth discussing the economic impacts of using mNGS and ddPCR methods to identify causative pathogens in BSIs. In China, currently, the cost of mNGS testing is approximately $450 per sample, $150 for ddPCR assays covering common isolated pathogens and AMR genes, and $60 for blood culture. Compared with traditional blood culture, mNGS and ddPCR methods are still comparatively expensive. However, recent studies have shown that the rapid and accurate identification of causative pathogens using nucleic acid detection methods is associated with improved mortality and reduced healthcare costs (Forrest et al., 2006; Perez et al., 2013). In a total of 221 episodes of sepsis, a mathematical prediction model demonstrated that the incremental cost using PCR assay was justified for patients with over 25% inadequate initial treatment, especially in the presence of high daily treatment cost and risk of severe complications from inadequate treatment (Lehmann et al., 2010). Therefore, mNGS and ddPCR are becoming cost-effective for improving the clinical outcome in patients with BSIs.

This study should be interpreted within the context of its limitations. First, the AMR genes could not be detected by mNGS testing in the present study due to a relatively low sequencing depth and the lack of a human-DNA depletion step. Second, in view of a great majority of the patients taking antimicrobial therapy prior to the ddPCR assay, whether the low pathogen DNA loads in blood samples resulted in a true BSI or were possibly derived from previous antimicrobial therapy could not be determined. Third, since our study had a relatively small sample size, with only 10 blood culture isolates identified, the results need to be further verified by expanding the sample size.

In conclusion, both ddPCR and mNGS have great potential in identifying causative pathogens of BSIs. The ddPCR assay is more rapid and sensitive for target pathogen identification than mNGS testing and has a certain advantage over mNGS testing in terms of its ability to detect AMR genes, whereas mNGS testing has a wider coverage of causative pathogens than ddPCR since the latter has a limited detection panel.

## Data Availability Statement

The datasets presented in this study can be found in online repositories. The names of the repository/repositories and accession number(s) can be found below: NCBI BioProject, accession no: PRJNA693402.

## Ethics Statement

The study protocol was approved by the Institutional Review Board and Ethics Committee (No. 2019KY002) of Zhejiang Provincial People’s Hospital. The patients/participants provided their written informed consent to participate in this study.

## Author Contributions

RS and BH conceived the study design, analyzed and interpreted the data, and drafted the manuscript. YT, ZS, and XY performed research and contributed analytic tools. XL, YZ, RZ, and JL participated in study design, acquired the data, and helped revise the manuscript. All authors contributed to the article and approved the submitted version.

### Conflict of Interest

The authors declare that the research was conducted in the absence of any commercial or financial relationships that could be construed as a potential conflict of interest.

## Footnotes

^1^https://www.ncbi.nlm.nih.gov/refseq

## Supplementary Material

Supplementary Figure 1 Distribution of pathogens detected exclusively by the mNGS method. Blue bars, viruses; orange bars, Gram-negative bacteria; green bars, Gram-positive bacteria; red bars, fungi. ddPCR, droplet digital PCR; mNGS, metagenomic next-generation sequencing.

Supplementary Table 1 Pathogens and AMR genes detection panels for ddPCR assay.

Supplementary Table 2 Comparison of the patients’ characteristics.
