# Supplementary figures and images for: A Comparison of Blood Pathogen Detection Among Droplet Digital PCR, Metagenomic Next-Generation Sequencing, and Blood Culture in Critically Ill Patients With Suspected Bloodstream Infections
Source: Front Microbiol. 2021 May 17;12:641202. doi: 10.3389/fmicb.2021.641202 (PMC8165239; doi:10.3389/fmicb.2021.641202)

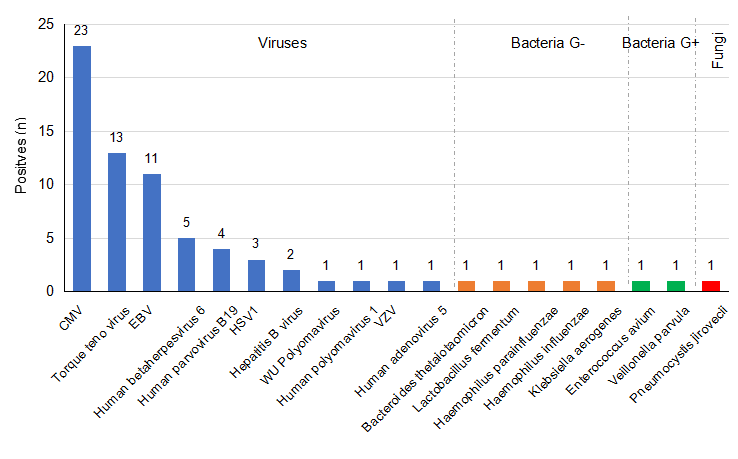

Supplement: Supplementary Figure 1 — Distribution of pathogens detected exclusively by the mNGS method. Blue bars, viruses; orange bars, Gram-negative bacteria; green bars, Gram-positive bacteria; red bars, fungi. ddPCR, droplet digital PCR; mNGS, metagenomic next-generation sequencing. [file Image_1.TIF]
