# Supplementary material for: A Comparison of Blood Pathogen Detection Among Droplet Digital PCR, Metagenomic Next-Generation Sequencing, and Blood Culture in Critically Ill Patients With Suspected Bloodstream Infections
Source: Front Microbiol. 2021 May 17;12:641202. doi: 10.3389/fmicb.2021.641202 (PMC8165239; doi:10.3389/fmicb.2021.641202)
Supplement: Supplementary Table 1 — Pathogens and AMR genes detection panels for ddPCR assay. [file Table_1.docx]

**Supplementary Table 1.** Pathogens and AMR genes detection panels for ddPCR assay.

| Assay panel | Target pathogens |
| --- | --- |
| PilotBac-1 | *A. baumannii, E. coli, K. pneumonia, P. aeruginosa,* |
| PilotBac-2 | *E. faecalis, E. faecium, S. aureus, S. pneumoniae* |
| PilotBac-3 | *S. capitis, S. haemolyticus, S. hominis, S. epidermidis* |
| PilotBac-4 | *E. cloacae, P. mirabilis, S. marcescens, S. maltophilia* |
| PilotFungi-1 | *C. albicans, C. glabrata, C. parapsilosis, C. tropicalis* |
| PilotAMR-1 | *bla_KPC_, mecA, vanA, vanB* |
