# Supplementary material for: A Comparison of Blood Pathogen Detection Among Droplet Digital PCR, Metagenomic Next-Generation Sequencing, and Blood Culture in Critically Ill Patients With Suspected Bloodstream Infections
Source: Front Microbiol. 2021 May 17;12:641202. doi: 10.3389/fmicb.2021.641202 (PMC8165239; doi:10.3389/fmicb.2021.641202)
Supplement: Supplementary Table 2 — Comparison of the patients’ characteristics. [file Table_2.docx]

**Supplementary Table 2.** Comparison of the patients’ characteristics

| Clinical characteristics | Blood culture positive  (n = 10) | ddPCR or NGS positive  (n = 25) | ddPCR and NGS negative  (n = 10) | *P* |
| --- | --- | --- | --- | --- |
| Age (years) | 65.8 ± 13.0 | 65.4 ± 13.9 | 66.3 ± 15.3 | 0.86 |
| Male, n (%) | 9 (90.0) | 9 (80.0) | 8 (80.0) | 0.77 |
| Use of vasoactive drugs, n (%) | 9 (90.0) | 17 (68.0) | 5 (50.0) | 0.15 |
| Mechanical ventilation, n (%) | 10 (100) | 21 (84.0) | 9 (90.0) | 0.39 |
| Acute kidney injury, n (%) | 4 (40.0) | 10 (40.0) | 5 (50.0) | 0.85 |
| Renal replacement therapy, n (%) | 4 (40.0) | 9 (36.0) | 5 (50.0) | 0.75 |
| Physical examinations |  |  |  |  |
| Temperature (°C) | 38.9 ± 0.88 | 38.6 ± 0.93 | 38.5 ± 1.01 | 0.53 |
| Systolic blood pressure (mmHg) | 80.6 ± 13.6 | 93.9 ± 15.6 | 96.2 ± 19.1 | 0.05 |
| Diastolic blood pressure (mmHg) | 42.1 ± 8.30 | 51.4 ± 12.0 | 48.5 ± 9.7 | 0.08 |
| Complete blood counts and blood biochemistry |  |  |  |  |
| Platelet counts, median (IQR) ×10^3^/μl | 58.5 (24.0 - 111) | 81 (39 - 191) | 106 (59 - 178) | 0.74 |
| White blood cell, median (IQR) ×10^3^/μl | 14.3 (7.46 - 21.3) | 9.68 (6.87 - 15.1) | 12.5 (8.56 - 17.0) | 0.29 |
| C reactive protein (mg/L), median (IQR) | 199 (125 - 320) | 167 (113- 243) | 76 (56 - 121) | 0.014 |
| Procalcitonin (μg/L), median (IQR) | 20.6 (3.08 - 31.0) | 3.40 (1.40 - 13.3) | 1.20 (0.50 - 2.30) | 0.023 |
| Serum creatinine (μmol/L), median (IQR) | 125.8 (97.0 - 157.8) | 117.2 (75.9 - 190.7) | 92.8 (68.3 - 126.2) | 0.31 |
| Serum lactate (mmol/L), median (IQR) | 2.70 (1.80 - 7.10) | 2.40 (1.80 - 3.80) | 1.90 (1.50 - 3.01) | 0.43 |
| SOFA score | 12.2 ± 3.39 | 11.2 ± 5.40 | 8.11 ± 5.25 | 0.18 |
| APACHE II score | 26.4 ± 5.71 | 24.5 ± 9.74 | 19.7 ± 8.92 | 0.23 |
| 28-day mortality, n (%) | 8 (80.0) | 14 (56.0) | 4 (40.0) | 0.19 |

IQR, interquartile range; SOFA, sequential organ failure assessment; APACHE II, acute physiology and chronic health evaluation II; ddPCR, droplet digital PCR; mNGS, Metagenomic next-generation sequencing.
